# Number of Components of the Metabolic Syndrome; Smoking and Inflammatory Markers

**DOI:** 10.5812/ijem.8403

**Published:** 2012-12-21

**Authors:** Tomoyuki Kawada, Toshiaki Otsuka, Tokiomi Endo, Yoichi Kon

**Affiliations:** 1 Department of Hygiene and Public Health, Nippon Medical School, Bunkyo-Ku, Tokyo, Japan; 2 Division of Health Evaluation & Promotion, Ota Memorial Hospital, Ota-city, Gunma, Japan

**Keywords:** Metabolic Syndrome, Inflammation, Smoking

## Abstract

**Background:**

The association between inflammatory markers and the combination of the smoking status plus a number of components of the metabolic syndrome was not fully evaluated in male Japanese subjects.

**Objectives:**

To demonstrate the association between inflammatory markers and the number of components of the metabolic syndrome by considering smoking status.

**Patients and Methods:**

A total of 3,017 male subjects (1,047 current smokers, 1,970 non-smokers) were included. Metabolic syndrome (MetS) was defined by the criteria of the National Cholesterol Education Program Adult Treatment Panel III. The smoking status was categorized in a binary manner into current smokers or non-smokers.

**Results:**

The geometric mean value of the serum CRP increased linearly as the number of components of MetS increased (P < 0.05). In contrast, the mean values of the total WBC, neutrophil, lymphocyte and monocyte counts showed peak values when the number of MetS components was 3 or 4. The log-transformed serum CRP levels and the WBC counts were significantly correlated with one another (P < 0.001), but Pearson’s correlation coefficient was under 0.3 for current smokers.

**Conclusions:**

Among several inflammatory markers, the serum CRP predominantly changed linearly as the number of MetS increased regardless of smoking status.

## 1. Background

Cigarette smoking and metabolic syndrome (MetS) are known risk factors for cardiovascular disease (1-3). Clinically, the risk of cardiovascular disease (CVD) increases with the progression of vascular inflammation (4), and cigarette smoking accelerates this pathological process.

Reports have suggested that the serum C-reactive protein (CRP) levels and white blood cell (WBC) counts are higher among current smokers than in never-smokers (5-7), and that the levels become greater with increasing number of cigarettes smoked per day (8, 9). However, consideration on the association among inflammatory markers, smoking and MetS component has not been fully conducted (10).

Ichihara et al. reported that elevated inflammatory indices such as CRP and WBC were associated with more coronary risk factors and poorer physical fitness (11), but CRP was measured by conventional latex immunoturbidimetric assay, not by highly sensitive CRP measurement. The methodological update is also required for the analysis.

## 2. Objectives

The authors conducted a cross-sectional study on the relationship between inflammatory markers including highly sensitive CRP and the number of MetS components by considering smoking status.

## 3. Patients and Methods

The authors previously described precise information on subjects in a preliminary study (12). Namely, the 3,269 male subjects were originally recruited to this survey. They attended voluntarily to the intensive health examination in Gunma prefecture, Japan, from the year 2008 to 2010. Subjects with coronary heart disease (n = 156) and/or 62 subjects with cerebrovascular disease, 44 subjects with serum CRP levels of ≥ 10mg/L, 4 subjects with WBC counts of ≥ 15,000/cmm or < 2,000 were excluded. Instead, 535 patients with receiving hypertension medication, 115 patients with receiving diabetes mellitus medication and/or 228 patients with dyslipidemia medication were included. Finally, 3,017 male subjects (1,047 smokers, 1,970 non-smokers) were included for the analysis.

Venous blood was collected following the patients had fasted overnight. Serum levels of triglycerides, high-density lipoprotein cholesterol (HDL), CRP and the fasting blood glucose were determined (AU2700, Olympus Co. Ltd., Japan). The lower detection limit of the serum CRP determination was 0.1 mg/L, and the intra-assay coefficient of variation was under 5%. Values of the serum CRP under the detection limit were recorded as 0.05 mg/L. The blood pressure was measured twice in all the participants (Nippon COLIN BP-103iII, Japan) and the values from the second measurement were adopted for the analysis. Waist circumference was measured at the level midway between the iliac crest and the 12th rib.

In accordance with the criteria of the US National Cholesterol Education Program Adult Treatment Panel III (NCEP-ATPIII) (13, 14), MetS was diagnosed when 3 or more of the following criteria were fulfilled: fasting blood glucose ≥ 100 mg/dL (5.6 mmol/L) or receiving treatment for diabetes mellitus, blood pressure ≥ 130/85 mm Hg (either value) or receiving antihypertensive drug treatment, plasma triglycerides ≥ 150 mg/dL (1.7mmol/L), plasma HDL < 40 mg/dL (1.0mmol/L), and waist circumference ≥85 cm (15). Each component of MetS was assigned a value of 1 when it was judged as being present, and 0 when it was judged as being absent. Smoking status of the subjects was classified as current- and non-smokers including never-smokers.

Informed consent was obtained from all the participants, and this study was approved by the Institutional Review Board at Ota General Hospital, Gunma Prefecture, Japan (July 17, 2010).

Two-way analysis of variance by the smoking status and number of MetS components, Fisher’s exact test and correlation analysis were performed using the SPSS version 16.0 (SPSS Inc Japan). The results are expressed as means and standard deviations (SD), except for parameters with a skewed distribution to the left, such as the triglycerides and CRP. P < 0.05 was adopted as the significance level.

## 4. Results

The 3,017 subjects were 51.6 ± 9.6 years old (range, 27 to 84 years). The percentage of current smokers was 34.7% (1047/3017). Percentages of smoking with and without MetS were 34.0% (326/958) and 35.0% (721/2059), respectively. There was no significant difference in percentage of smoking between MetS and non-MetS subjects. Percentages of smoking in 30s, 40s, 50s, 60s and 70s were 43.8% (326/958), 42.5% (388/912), 33.0% (372/1127), 23.6% (133/563) and 11.0% (8/73), respectively.

The mean values and SDs of the levels of the inflammatory markers in the subjects are listed in Table 1. Two-way analysis of variance was conducted on the inflammatory markers. There was no interaction between the smoking status and the number of MetS components with the WBC, neutrophil, lymphocyte or monocyte counts, or with the log-transformed serum CRP levels, respectively. Among several inflammatory markers, the geometric mean of the serum CRP increased linearly as the number of MetS components increased irrespective of smoking status (P < 0.05).

The Pearson’s moment correlation coefficients (CC) among the WBC, neutrophil, lymphocyte and monocyte counts, and the log-transformed serum CRP stratified by the smoking status are listed in Table 2. The log-transformed serum CRP levels and the WBC counts were significantly correlated with one another (P < 0.001). However the CC was under 0.3 for current smokers. In the case of non-smokers, the CC became higher, except for the count of lymphocytes.

**Table 1:** Means and Standard Deviations of Inflammatory Markers Stratified by the Smoking Status and Number of MetS Components

Numbers of MetS	WBC, /cmm	Neutrophil, /cmm	Lymphocyte, /cmm	Monocyte, /cmm	CRP, mg/L
	Current Smokers n=1.047 a					
**0 (208)**	6330, 1640	3590, 1280	1970, 560	360, 120	0.35, 2.97
**1 (280)**	6500, 1750	3700, 1340	2030, 560	360, 100	0.40, 2.88 b
**2 (233)**	6800, 1540 b	3890, 1210	2090, 560	380, 110	0.60, 2.89 b
**3 (185)**	7350, 1950 b	4270, 1590 b	2260, 630 b	400, 120 b	0.69, 2.90 b
**4 (96)**	7260, 1690 b	4040, 1290 b	2340, 600 b	400, 130 b	0.88, 2.55 b
**5 (45)**	6990, 1650	3940, 1280	2210, 610 b	380, 90	1.07, 2.37 b
	**Nonsmokers Including Never Smokers and Ex-smokers n=1,970 a**				
**0 (366)**	5100, 1230	2840, 910	1640, 450	280, 90	0.27, 2.83
**1 (489)**	5480, 1250 b	3060, 1010 b	1780, 470 b	300, 80	0.34, 2.82 b
**2 (483)**	5660, 1320 b	3130, 1020 b	1860, 520 b	310, 90 b	0.49, 2.85 b
**3 (362)**	5910, 1300 b	3280, 940 b	1930, 510 b	320, 100 b	0.62, 2.65 b
**4 (193)**	6050, 1520 b	3390, 1180 b	1950, 540 b	320, 100 b	0.64, 2.80 b
**5 (77)**	5980, 1440 b	3360, 1100 b	1910, 500 b	340, 100 b	0.69, 2.76 b

Abbreviations: MetS; metabolic syndrome, WBC; white blood cell, CRP; C-reactive protein

^a^There was a significant difference of mean value for each variable by the analysis of variance

^b^Dunnett’s multiple comparison was conducted, and was described when P < 0.05 was recognized compared with control group (number of MetS = 0).

**Table 2:** Pearson’s Moment Correlation Coefficients Among Inflammatory Markers

Current smokers	WBC	Nonsmokers Including Never Smokers and Ex-Smokers n=1.970			
		Neutrophil	Lymphocyte	Monocyte	Log 10(CRP)
**WBC**	-	0.902	0.584	0.638	0.337
**Neutrophil**	0.92	-	0.202	0.543	0.309
**Lymphocyte**	0.602	0.261	-	0.31	0.163
**Monocyte**	0.654	0.533	0.424	-	0.295
**Log10 (CRP)**	0.285	0.255	0.171	0.238	-

Abbreviations are listed also in Table 1.

## 5. Discussion

In this study, CRP increased steadily with increasing the number of MetS components among several inflammatory markers, and this increase was not modified by smoking. In addition, CRP was not highly correlated with other inflammatory markers.

Former studies have shown elevated serum CRP levels in current smokers as compared with those in non-smokers (5, 7-9). The authors observed no interaction between the smoking status categorized in a binary fashion and the number of MetS components.

Several studies have reported the positive relationship between smoking and elevated inflammatory indices, and smoking increases genetic expression and serum concentration of interleukin-6 (IL-6) (16). As one of the pathways, smoking induces IL-6 from visceral adipose tissue and IL-6 accelerates CRP production by hepatocytes (17). Combination with other cytokines should also be evaluated to elucidate the meaning of CRP.

Ridker et al. have proposed CRP as an indispensable factor for the prediction of cardiovascular risk with the advantage that CRP measurement is inexpensive, standardized and available worldwide (18). They recently recommended the use of CRP as an inflammatory marker in the fields of clinical cardiology and prevention by meta-analysis (19). From the linear relationship between CRP and the number of MetS regardless of smoking status, we have suggested the use of CRP as an inflammatory biomarker for the prevention of CVD.

There are some limitations in our study. Firstly, smoking status was only classified in a binary fashion. Therefore, the dose-response relationship between the smoking status and inflammatory markers could not be evaluated. Secondly, we could not conclude the cause-effect relationship between the inflammatory markers, smoking status and number of components of MetS, because of the cross-sectional nature of this study.

The authors conclude that current smokers show higher serum levels of CRP and WBC counts, including the WBC subtype counts. However, the trend towards increase in the values with the number of components of MetS differed between the two parameters. The relationship between the serum CRP and the smoking status appeared to be weaker than the relationship between the serum CRP and the risk of MetS, and the authors speculate that the time-course behaviors of inflammatory markers such as the serum CRP and peripheral blood WBC count differ in relation to the progression of atherosclerosis. Further study is recommended to explore the association between MetS and inflammatory markers including CRP and WBC counts.
